# Immune activation of vaginal human Langerhans cells increases susceptibility to HIV-1 infection

**DOI:** 10.1038/s41598-023-30097-x

**Published:** 2023-02-25

**Authors:** Nienke H. van Teijlingen, Julia Eder, Ramin Sarrami-Forooshani, Esther M. Zijlstra-Willems, Jan-Paul W. R. Roovers, Elisabeth van Leeuwen, Carla M. S. Ribeiro, Teunis B. H. Geijtenbeek

**Affiliations:** 1 Amsterdam UMC Location Academic Medical Center, Experimental Immunology, Meibergdreef 9, Amsterdam, The Netherlands; 2 Amsterdam Institute for Infection & Immunity, Amsterdam, The Netherlands; 3 ATMP Department, Breast Cancer Research Center, Motamed Cancer Institute, ACECR, P.O. BOX, Tehran, 15179/64311 Iran; 4 Amsterdam UMC Location Academic Medical Center, Obstetrics and Gynaecology, Meibergdreef 9, Amsterdam, The Netherlands

**Keywords:** Translational immunology, Mucosal immunology, HIV infections

## Abstract

Vaginal inflammation increases the risk for sexual HIV-1 transmission but underlying mechanisms remain unclear. In this study we assessed the impact of immune activation on HIV-1 susceptibility of primary human vaginal Langerhans cells (LCs). Vaginal LCs isolated from human vaginal tissue expressed a broad range of TLRs and became activated after exposure to both viral and bacterial TLR ligands. HIV-1 replication was restricted in immature vaginal LCs as only low levels of infection could be detected. Notably, activation of immature vaginal LCs by bacterial TLR ligands increased HIV-1 infection, whereas viral TLR ligands were unable to induce HIV-1 replication in vaginal LCs. Furthermore, mature vaginal LCs transmitted HIV-1 to CD4 T cells. This study emphasizes the role for vaginal LCs in protection against mucosal HIV-1 infection, which is abrogated upon activation. Moreover, our data suggest that bacterial STIs can increase the risk of HIV-1 acquisition in women.

## Introduction

HIV-1/AIDS remains the leading cause of death for African women of reproductive age and these women are more than 2 times as likely to become HIV-1 infected compared to their male peers^1,2^. In women, the major route of infection is sexual transmission via the vaginal mucosa^3^. Vaginal mucosal tissue protects women against environmental cues such as invading pathogens, mechanical stress (i.e. intercourse), and constant exposure to commensal microbiota^4,5^. Vaginal mucosa can be divided into two major compartments; the epithelial layer and the subepithelial lamina propria. The epithelial layer consists of non-keratinized stratified squamous epithelial cells and harbours only a few specific immune cells. In contrast, the lamina propria is composed of connective tissue and is rich in immune cells^6,7^.

The vaginal epithelium is the first compartment to become exposed to HIV-1 upon sexual transmission and constitutes a physical barrier to viral particles by its multi-layers, tight junctions, and secreted mucus. Although not as extensively as the lamina propria, the epithelial layer is equipped with various local immune players^6,8^. Besides antimicrobial peptides (i.e. LL-37 and defensins) and immunoglobulins (i.e. IgA and IgG), the epithelial layer contains T cells and Langerhans cells (LCs)^6,8,9^. LCs embody a subtype of antigen presenting cells (APCs) that play a central role in mucosal immunology and distinctively express the C-type lectin receptor langerin^10,11^. In the vaginal epithelium, LCs are resident immune cells that express HIV-1 entry receptors^12–15^. LCs are part of the dendritic cell lineage but also share close traits with macrophages through a common precursor^16^. Moreover, LCs repopulate locally independent of blood circulation^17^. During HIV-1 infection, LCs can be both protective detrimental^18^. Immature LCs exert anti-viral properties in vitro as they restrict infection with low doses of HIV-1 through capture by langerin and subsequent internalization and TRIM5α/autophagy-mediated degradation^19–21^. However, LCs can also be productively infected by HIV-1 through their CD4 and CCR5 receptors^15,22^. Moreover, recent studies identified new cell populations that are closely related to “classic” LCs^23–25^. Vaginal epithelial dendritic cells (VEDCs) are characterized by their CD1a and langerin expression but harbor no Birbeck granules^23^. Epidermal CD11c + DCs are CD1a positive but express low levels of langerin^23,25^. Compared to classic LCs, these cells are more permissive to HIV-1 infection and transmission^23,25^. While inflammatory responses by LCs and other immune cells are beneficial and required to effectively eliminate sexually transmitted infections (STIs), vaginal inflammation prior to HIV-1 exposure paradoxically increases the risk of HIV-1 acquisition^26–29^. Local vaginal inflammation not only hampers the barrier function of vaginal epithelium, but also activates LCs leading to attenuation of their protective role in HIV-1 infection^14,20,30^. Inflammatory stimuli increase the ability of skin LCs to capture and transmit HIV-1 while also enhancing the replicative potential of HIV-1 in LCs^30^. Interestingly, agonists to the Toll-like receptor (TLR) 2 enhance susceptibility of LCs to HIV-1, suggesting that gram + bacteria might be responsible for enhanced infection and transmission of HIV-1^31^. Recently, we have shown that the vaginal bacterium *Prevotella timonensis* enhances HIV-1 capture as well as transmission by LCs, providing further evidence that bacterial species can influence HIV-1 susceptibility of target cells^32^. Moreover, elevated levels of pro-inflammatory cytokines can recruit activated CD4 T cells from the lamina propria, supplying additional HIV-1 target cells^33^.

Our current study uses physiologically important immature human LCs freshly isolated from vaginal mucosa and shows that they restrict HIV-1 infection and pose a barrier to sexual HIV-1 transmission in women. In contrast, mature vaginal LCs were efficiently infected by HIV-1 and largely transmitted HIV-1 to CD4 T cells. Notably, exposure to bacterial, but not viral, TLR ligands enhanced HIV-1 infection and transmission of immature vaginal LCs. Thus, this study supports a role for human vaginal LCs in protection against HIV-1 and suggests that activation of vaginal LCs by bacteria increases sexual HIV-1 susceptibility in women.

## Results

### Vaginal LC purification

**Figure 1:**
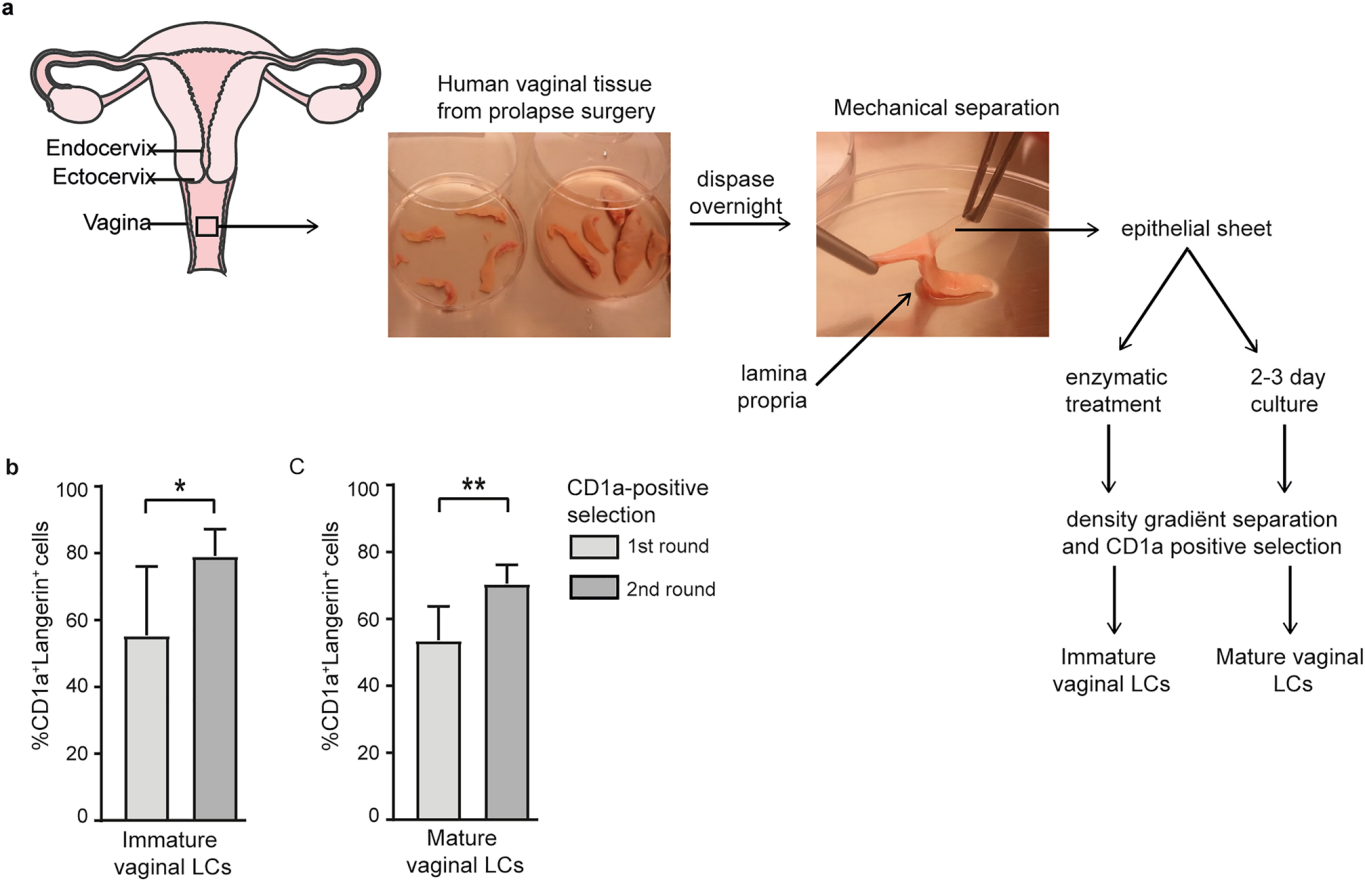
Vaginal LC purification. (**a**) schematic model and pictures of isolation procedures used to obtain immature and mature vaginal LCs from primary human vaginal tissue. Images were adopted from Servier Medical Art by Servier (http://www.servier.com/Powerpoint-image-bank) and modified by the authors under the following terms: CREATIVE COMMONS Attribution 3.0 Unported (CC BY 3.0); (**b,c**) vaginal LC purity (% CD1a + /Langerin^high^ cells) after immature ((**b**), N = 7) and mature ((**C**), N = 6) isolation procedure. *p < 0,05, **p < 0.01, two-tailed *t-*test, data are mean ± SD.

To obtain immature and mature LCs from vaginal epithelium, a combination of mechanical and enzymatic methods was used (Fig. 1a). Epithelial sheets were separated from the lamina propria and submucosal stroma using surgical scissors and subsequent overnight incubation with the enzyme Dispase II. Smooth separation after Dispase II incubation could only be secured when stromal layers were thin and cut strips did not exceed a width of 7 mm. Immature LCs were immediately isolated from epithelial sheets using enzymatic digestion. For mature vaginal LCs, epithelial sheets were cultured for several days and emigrated CD1a + were harvested. Both immature and mature vaginal LCs were purified using density gradient isolation and subsequent positive CD1a selection with MACS. After two rounds of CD1a selection, the immature isolation protocol resulted in an immature LC purity, defined as CD1a + and langerin^high^ expressing cells) of 79.1% (ranging 63.9–90.0%, Fig. 1b and Supplemental Fig. 1a). One CD1a selection round resulted in 55.5% immature LC purity (range 23.7–78.0%, Fig. 1b). For the mature LC model, cell purity accounted for 53.6% (1st CD1a selection round, range 37.8–68.5%, Fig. 1c) and 70.5% (2nd CD1a selection round, range 63.3–73.3%, Fig. 1c). Isolation of vaginal cells as described in Fig. 1 resulted in a consistent purity of CD1a positive cells stably above 85% (Supplemental Fig. 1b). The CD1a positive cells highly expressed HLA-DR, CD11b, E-cadherin and Mucin-1. The co-purified/isolated CD1a negative fraction was slightly enriched for CCR5, but no particular cell type could be identified as specific markers for vaginal epithelial cells were not available (data not shown). Both enzymatically isolated and emigrated CD1a + cells expressed langerin, suggesting that these are LCs. Thus, LCs were purified from vaginal epithelium using a combination of mechanical and enzymatic methods, resulting in CD1a + /langerin^high^ purities.

### Emigrated mature vaginal LCs express lower levels of langerin and higher levels of CD86

**Figure 2:**
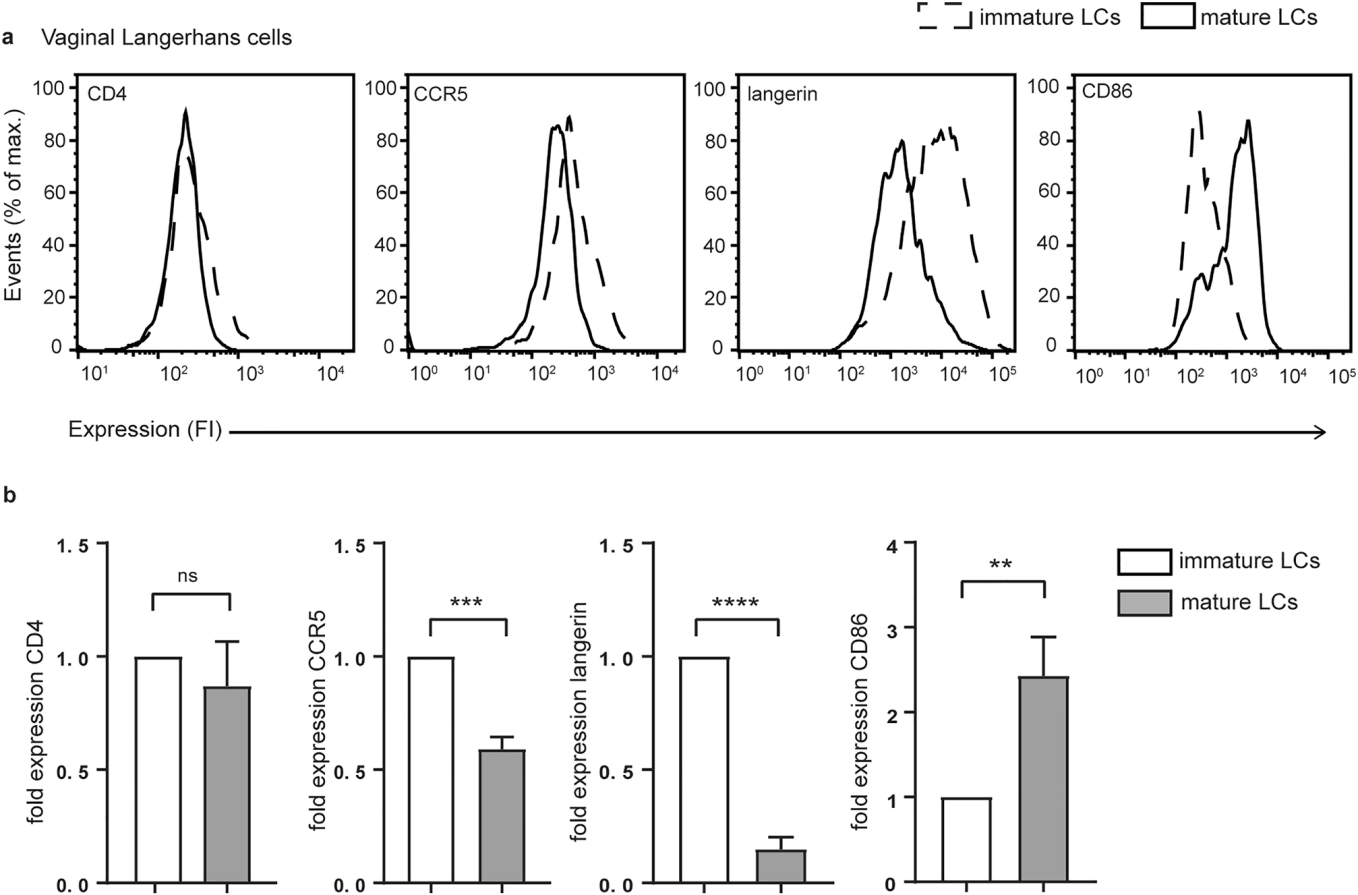
Immature and mature vaginal LCs diplay a differential phenotype. (**a**) representative plots of the expression of LC receptors involved in the interaction with HIV-1 (CD4, CCR5, langerin) and T cells (CD86) on immature and mature vaginal LCs as determined by flow cytometry. ((**b**), N = 3) pooled data from 3 separate donors of immature and mature vaginal LCs (CD1a +) for the expression of CD4, CCR5, langerin and CD86. **p < 0.01, ***p < 0.001, unpaired *t-*test, data are mean ± SD.

Both immature and mature CD1a + LCs were analysed for expression of CD4, CCR5, langerin and CD86 by flow cytometry. As described previously, HIV-1 receptor CD4 and HIV-1 co-receptor CCR5 were detected on immature and mature vaginal LCs (Fig. 2a,b)^12,34^. CCR5 was higher expressed by mature LCs, whereas CD4 expression was similar on both immature and mature LCs isolated from the same vaginal mucosa explants (Fig. 2). Both immature and mature LCs expressed langerin, a C-type lectin receptor involved in HIV-1 binding and TRIM5α/autophagy-mediated degradation^20,21^ (Fig. 2). Notably, langerin expression was substantially decreased in mature vaginal LCs compared to immature LCs (Fig. 2), similar to what we have previously observed in skin derived LCs^35^. Mature LCs expressed higher levels of co-stimulatory molecule CD86 (Fig. 2), indicating the increased capacity to prime naïve T cells. Taken together, vaginal LCs express several surface receptors involved in the initial interaction between LCs and HIV-1.

### Vaginal LCs express functional viral and bacterial TLRs

**Figure 3:**
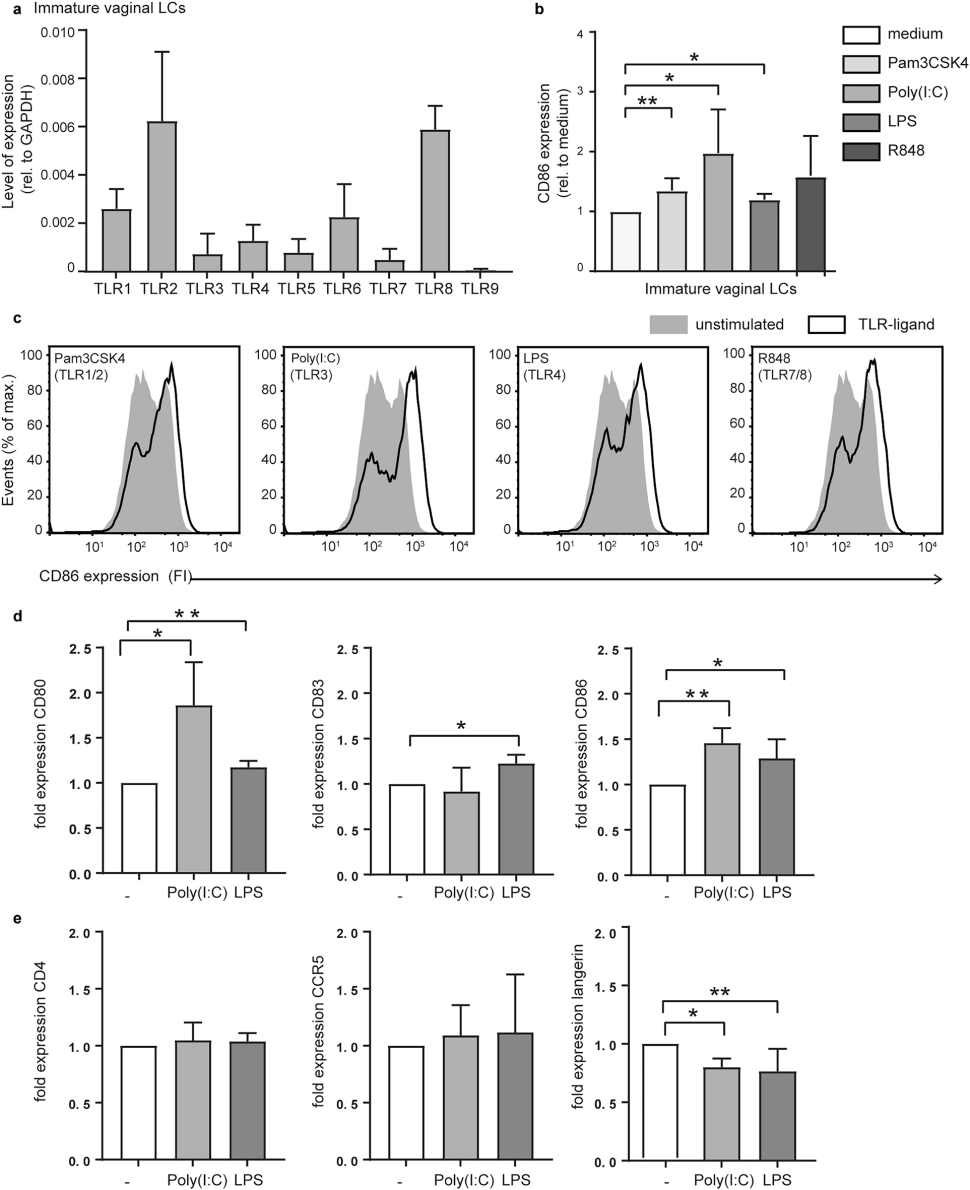
Vaginal LCs express functional viral and bacterial TLRs. (**a**) TLR expression levels of immature vaginal LCs (CD1a + , Langerin^high^) as determined by RT-PCR (N = 4); (**b**) relative maturation (MFI CD86 ‘TLR-ligand’/MFI CD86 ‘medium’) of immature LCs exposed to TLR-ligands (N = 4–6); *p < 0,05, **p < 0.01, two-tailed *t-*test, data are mean ± SD; (**c**) representative histograms of CD86 upregulation on immature vaginal LCs (CD1a + , Langerin^high^) in response to TLR-ligands as determined by flow cytometry (unstimulated—filled grey histogram; TLR-ligand—black line). ((**d**), N = 5, N = 4 and N = 5 respectively) pooled data of expression of CD80, CD83 and CD86 on seperated donors after stimulation with either LPS or Poly(I:C); ((**e**), N = 6) pooled data of separate donors for expression of CD4, CCR5 and langerin; *p < 0,05, **p < 0.01, two-tailed *t-*test, data are mean ± SD.

To investigate the ability of vaginal LCs to respond to bacterial and viral ligands, we determined their TLR expression profile. Vaginal LCs expressed all major TLRs, except TLR9 (Fig. 3a). Strikingly, TLR4 could be detected, whereas epidermal LCs lack the expression of this bacteria-sensing TLR^36^. Importantly, we sorted immature vaginal LCs into CD1a positive and CD1a negative fractions and only detected TLR4 on the CD1a positive cells (Supplemental Fig. 1c). To test the functionality of TLRs expressed by vaginal LCs, we exposed immature vaginal LCs (CD1a + and langerin^high^) to TLR ligands and analysed upregulation of co-stimulatory molecule CD86 as measure of TLR activation. Stimulation of immature vaginal LCs with bacterial ligands for TLR1/2 (Pam3CSK4) or TLR4 (LPS) resulted in a significantly increased expression of CD86 (Fig. 3b,c). The most extensive increase in CD86 expression was induced by viral TLR3 ligand Poly(I:C) (Fig. 3b,d). Levels of CD86 expression after stimulation with these activating TLR ligands were similar to the level of CD86 expression of emigrated mature LCs (Fig. 2b). TLR7/8 ligand R848 induced CD86 upregulation similar to Poly(I:C) but did not reach significance (*p-*value = 0.186). When stimulating the cells with Lipid A, key domain of LPS^37^, we observed similar upregulation of CD86 expression compared to LPS (Supplemental Fig. 1d). Furthermore, co-treatment with Polymyxin B sulfate, a potent antibiotic used to inhibit LPS^38^, decreased the LPS-induced CD86 expression (Supplemental Fig. 1d), supporting that LPS moiety is responsible for this cell maturation. Next, we wanted to examine the full co-stimulatory potential of vaginal LCs after TLR triggering. In addition to CD86, vaginal LCs were assessed for their ability to upregulate co-stimulatory molecules CD80 and CD83 in response to stimulation with Poly(I:C) and LPS (Fig. 3d). CD80 was upregulated both after Poly(I:C) and LPS stimulation, in accordance with CD86 (Fig. 3d). However, the expression of CD83 was similar between immature and mature LCs (Fig. 3d), suggesting these LCs are not fully matured but able to present antigens. We also investigated whether receptors important for HIV-1 infection of LCs, i.e. CD4, CCR5, and langerin, were affected by LC activation through TLRs (Fig. 3e). Neither CD4 nor CCR5 were affected by TLR stimulation with Poly(I:C) or LPS (Fig. 3e). These results contrast with CCR5 downregulation we observed upon LC maturation through migration (Fig. 2a,b), suggesting that activation after TLR stimulation induces a different LC phenotype. Importantly, langerin was significantly downregulated after TLR stimulation (Fig. 3e). Taken together, these results indicate that immature vaginal LCs harbour functional TLRs and therefore can respond to bacteria as well as viruses. Moreover, stimulation with TLR ligands leads to an activated LC phenotype that can prime LCs for antigen presentation as well as support HIV-1 infection.

### Immature vaginal LCs efficiently capture HIV-1 gp120 via langerin

**Figure 4:**
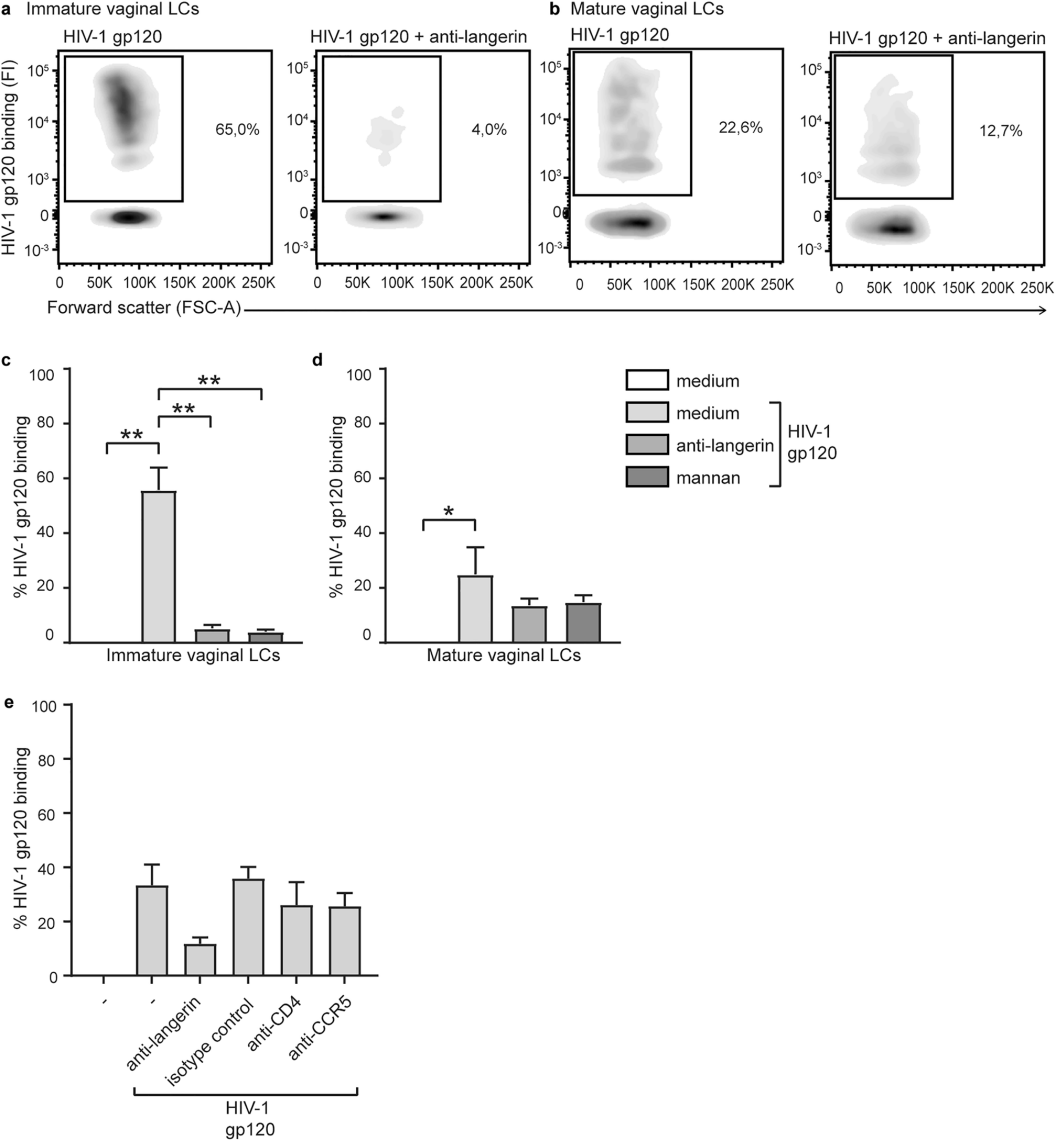
Immature vaginal LCs efficiently engage HIV-1 through langerin. (**a,b**) representative flow cytometry plots of HIV-1 gp120 binding to immature (**a**) and mature (**b**) vaginal (CD1a + , Langerin^high^) left untreated or pre-treated with anti-langerin; (**c,d**) pooled HIV-1 gp120 binding data (% HIV-1-gp120 +) of immature ((**c**), N = 3) and mature ((**d**), N = 3) vaginal LCs pre-treated with medium, anti-langerin, or mannan; *p < 0,05, **p < 0.01, two-tailed *t-*test; ((**e**), N = 2) GP120 binding (% HIV-1-gp120 +) on immature vaginal LCs in the presence or absence of anti-langerin (10E2), anti-DC-SIGN (D1, isotype control), anti-CD4 or anti-CCR5 blocking antibody; data are mean ± SD.

We next investigated the interaction of HIV-1 with immature and mature vaginal LCs using a fluorescent HIV-1 gp120-coated bead-binding assay. HIV-1 gp120 efficiently interacted with immature vaginal LCs, resulting in ~ 60% binding (Fig. 4a,c). HIV-1 gp120 binding to immature vaginal LCs was significantly decreased by a langerin-blocking antibody as well as by mannan, a mannosylated carbohydrate structure known for its binding to langerin. Mature LCs have a decreased langerin expression (Fig. 2b) and captured HIV-1 gp120 less efficiently (~ 20%, Fig. 4b,d) compared to immature LCs. Neither anti-langerin nor mannan could significantly block HIV-1 gp120 binding to mature vaginal LCs. Moreover, HIV-1 gp120 binding to immature vaginal LCs was only marginally decreased after blocking CD4 or CCR5 in comparison to the decrease observed with anti-langerin (Fig. 4e), suggesting that these receptors are less involved in the binding of HIV-1 to immature vaginal LCs. As expected, anti-DC-SIGN antibodies did not decrease binding. These results strongly suggest that langerin expressed on immature vaginal LCs efficiently captures HIV-1 gp120, and thereby considered a major HIV-1 binding receptor on LCs^20^. However, mature LCs have decreased langerin expression (Fig. 2b), suggesting that other receptors (i.e. CD4 and CCR5) play a role in virus binding and subsequent infection.

### Mature vaginal Langerhans cells become highly infected by HIV-1 and bacterial TLR ligands abrogate HIV-1 restriction in immature vaginal LCs

**Figure 5:**
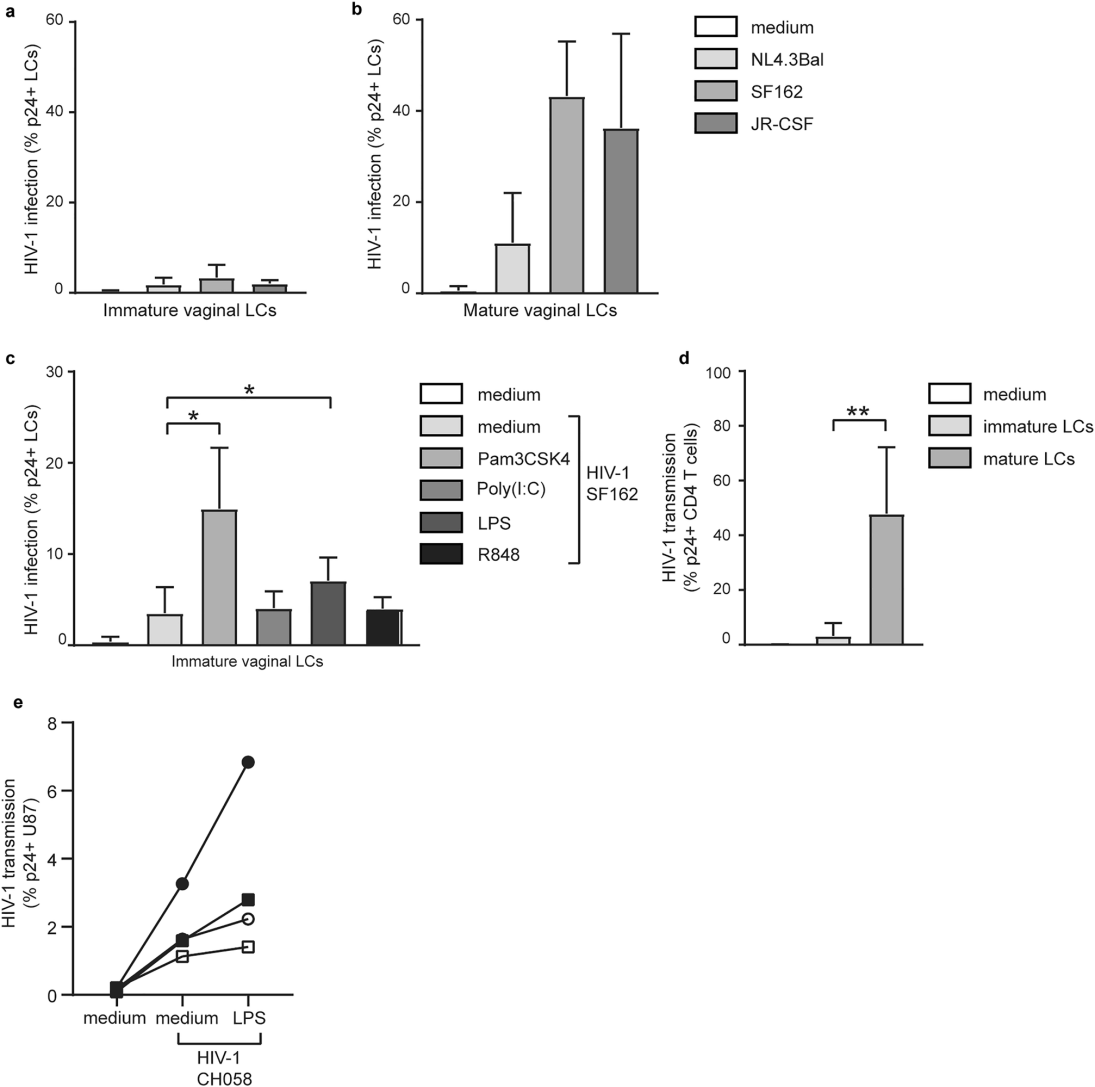
Bacterial TLR-ligands increase HIV-1 infection in vaginal LCs and activated vaginal LCs transmit HIV-1 to target cells. (**a,b**) HIV-1 (NL4.3-Bal, SF162, JR-CSF) infection of immature ((**a**), N = 3–10) and mature ((**b**), N = 2–5) vaginal LCs (CD1a + , langerin^high^) as measured by double staining for intracellular p24 and CD1a, and analyzed by flow cytometry; (**c**) HIV-1 (SF162) infection in immature vaginal LCs pre-stimulated with TLR-ligands, N = 4–7 *p < 0,05, two-tailed *t-*test, (**d**) HIV-1 (SF162) transmission from immature and mature vaginal LCs to CD4 T cells after co-culture, N = 3–5, **p < 0.01, two-tailed *t-*test, ((**e**), N = 4) HIV-1 transmitted/founder virus (CH058) transmission by immature vaginal LCs to CD4/CCR5 expressing U87 cells with or without prior stimulation or LPS for 30 min, data are mean ± SD.

LCs form a protective barrier against HIV-1 infection^10,19–21^. As shown for skin-derived LCs, the antiviral properties can be attenuated when LCs are subjected to immune activation; instead of targeting HIV-1 for degradation, LCs become productively infected^19–21^. Here we investigated the levels of HIV-1 infection of immature as well as of mature vaginal LCs (CD1a + and langerin^high^) after a 5-day exposure to various HIV-1 strains. Immature vaginal LCs showed low levels of HIV-1 infection after exposure to different HIV-1 strains NL4.3-Bal, SF162, and JR-CSF. Infection levels (% p24 + of CD1a + cell fraction) varied from 1.3 to 6.6% (Fig. 5a). In contrast, higher levels of HIV-1 infection were observed in mature CD1a + vaginal LCs (Fig. 5b). HIV-1 infection levels of mature cells varied more compared to immature vaginal LCs, ranging from 4.3 to 51.7%, and were clearly higher for the PBMC produced HIV-1 strains SF162 and JR-CSF (~ 43.2 and ~ 36.3%) than for NL4.3-Bal (~ 11.1%).

Additionally, vaginal immature LCs were stimulated with various TLR ligands to investigate their influence on the HIV-1 restrictive function of immature vaginal LCs. Immature vaginal LCs were stimulated overnight with Pam3CSK4, Poly(I:C), LPS, and R848, and subsequently infected with HIV-1 (SF162). Notably, the bacterial ligand Pam3CSK4 (TLR1/2) and LPS (TLR4) specifically increased the infection levels in vaginal immature LCs (Fig. 5c). Stimulation with viral ligands Poly(I:C) (TLR3) and R848 (TLR 7/8), which efficiently activated immature vaginal LCs (Fig. 3b,c), did not result in increased HIV-1 infection compared to unstimulated LCs (Fig. 5c). Infection levels in Pam3CSK4- and LPS-stimulated LCs (max 3.6-fold increase), however, did not reach the magnitude of HIV-1 infection observed in the mature vaginal cell model (12.5-fold increase; Fig. 5b). These data indicate that immature vaginal LCs are poorly susceptible to R5-tropic HIV-1, whereas mature vaginal LCs are efficiently infected by R5-tropic HIV-1 strains. Moreover, our findings suggest that bacterial co-infections increase HIV-1 susceptibility by abrogating the restriction of HIV-1 infection in immature vaginal LCs.

### Mature vaginal Langerhans cells effectively transmit HIV-1 to target cells

Next, we investigated the ability of both immature and mature vaginal LCs to transmit HIV-1 to target cells. First, vaginal LCs were exposed to HIV-1 for 2 days, allowing productive infection of LCs, then washed extensively and subsequently co-cultured with PHA/IL2-activated CD4 T cells in a 1:2 ratio for 3 days. Transmission of HIV-1 (SF162) by immature vaginal LCs was low (~ 3.3%, Fig. 5d). In contrast, mature vaginal LCs efficiently transmitted HIV-1 to CD4 T cells, resulting in infection levels of ~ 47.3% in CD4 T cells after co-culture. Thus, mature vaginal LCs are productively infected with HIV-1 resulting in abundant HIV-1 transmission to target CD4 T cells. During sexual transmission of HIV-1, multiple viral strains are present in the genital tract. However, typically only one strain, termed transmitted/founder (T/F) virus, establishes infection^39,40^. Vaginal LCs are more susceptible to T/F viruses than to their chronic HIV-1 counterparts, exhibiting increased infection^41^. We therefore incubated LPS-activated vaginal LCs with the HIV-1 T/F virus CH058 and co-cultured them with susceptible target cells and determined transmission after several days (Fig. 5e). Although we observed donor differences, LPS-activated LCs showed a trend in increased HIV-1 T/F transmission, indicating that activation of vaginal mucosa by bacteria can enhance HIV-1 T/F transmission by vaginal LCs.

## Discussion

HIV-1/AIDS is still a global health problem and sexual transmission is the major route of infection^2,42^. Vaginal STIs compose a risk for acquisition of HIV-1 and previous studies point out immune activation as being the mediating factor^26–29^. More specifically, TLR ligation has been suggested to affect susceptibility of LCs in vaginal mucosa and enhance sexual transmission of HIV-1^31^.

Here we have investigated primary human vaginal LCs in the context of immune activation. Skin-derived LCs do not express TLR4^36,43^ and, notably, here we show that vaginal LCs express TLR4 and this affects HIV-1 infection. Immune activation in ex vivo culture of immature mucosal sheets or direct activation of immature vaginal LCs by bacterial TLR ligands, including LPS, enhanced HIV-1 infection as well as transmission to CD4 T cells. These data indicate that vaginal LCs might be more susceptible to HIV-1 in the presence of gram-negative bacteria observed in bacterial vaginosis.

LCs are considered a potential target for HIV-1 as they express entry receptors CD4 and CCR5, as shown in this report and previous studies^12,34^. HIV-1 binding to CD4 and CCR5 leads to fusion of the viral envelope with the target cell membrane. However, expression of langerin by LCs forms an infection barrier; langerin efficiently captures HIV-1 and targets the virus to a TRIM5α/autophagy-mediated degradation process^20,21^. Viral scavenging by langerin is thought to prevent HIV-1 infection of LCs but also prevents infection of surrounding cells. This protective effect depends on various factors such as viral load as well as expression of langerin. Therefore, at high concentrations, LCs can become infected by HIV-1. Importantly, while immature monocyte-derived LCs are poorly susceptible to HIV-1 infection, the chance for infection increases in their mature counterparts^44^. We show that immature vaginal LCs, directly isolated from fresh vaginal tissue, are poorly susceptible to HIV-1 infection. This is in accordance with studies from our group using immature LCs isolated from skin^14,20^ or vaginal mucosa^41^. As well as studies on vaginal explants that report no productive HIV-1 infection in vaginal LCs^9,45^. In line with these studies, we detected low HIV-1 infection in immature vaginal LCs. However, we observed high levels of infection in vaginal LCs that were matured through ex vivo culturing prior to HIV-1 exposure. This increased HIV-1 susceptibility might be explained by decreased expression of langerin, as we observed lower levels of langerin expression and a decreased capacity to bind gp120 coated beads by mature LCs compared to their immature counterparts. Interestingly, we still observe some gp120 binding to mature LCs after langerin block, suggesting that other receptors might be involved. Our data suggest that CD4 and CCR5 are not involved in the gp120 binding as antibodies against these receptors do not abrogate gp120 binding by immature LCs. It is possible that heparan sulfate proteoglycans are involved in the remaining gp120 binding by mature LCs^35^.

Recently, new subsets of langerin expressing cells were described in the skin and vaginal mucosa^23–25^. In the vaginal mucosa, CD1a + and langerin+ epithelial DCs were suggested to become infected by HIV-1 due to their lack of Birbeck granules^23^ while epidermal CD11c + DCs described in the epidermis and anogenital tissues were more susceptible to HIV-1 than LCs^25^. With our isolation method we obtained cells that expressed high levels of both CD1a and langerin while also expressing CD4 and CCR5. High expression of langerin and low susceptibility to HIV-1 suggests that we are looking at classic LCs. Another LC subset, termed LC2, was recently identified in the epidermis and differs phenotypically and functionally from classical LCs (LC1)^24^. As of now, there is no evidence of an LC2 population in vaginal mucosa and further research into their localization is needed.

LCs from skin do not express TLR4 and are unresponsive to LPS^36,46^. As human skin is an extensive and important organ, this unresponsiveness might be a mechanism contributing to the tolerance to bacterial commensals which prevents unnecessary inflammation^46^. Strikingly, our data strongly suggest that vaginal LCs are fully equipped with TLR1-8, including TLR4. Whereas, we did not observe any TLR4 expression in CD1a negative sorted vaginal cell fraction nor skin immature LCs (Supplemental Fig. 1c and data not shown). These data imply that vaginal LCs are able to recognize vaginal co-infections caused by viruses as well as bacteria.

By exposing immature vaginal LCs to TLR ligands we obtained an activated phenotype with increased levels of co-stimulatory molecules CD80 and CD86. Interestingly, CD83 did not increase in expression upon stimulation with either TLR ligand, suggesting the cells are not fully matured but activated and primed for antigen presentation. Notably, we observed stronger downregulation of langerin in emigrated mature LCs, suggesting that the TLR-activated LCs are less activated than mature LCs. This is further supported by finding that mature LCs were more efficient in HIV-1 transmission than TLR-activated LCs.

Only bacterial TLR ligands Pam3CSK4 and LPS increased HIV-1 infection in immature vaginal LCs. Strikingly, Poly(I:C) exposure resulted in high LC activation, but low HIV-1 infection of vaginal LCs, in line with previous reports in LCs from skin and in a monocyte-derived LC model^31^. Viral TLR3 ligand Poly(I:C), might induce an antiviral interferon response in vaginal LCs, which further preserves the anti-HIV-1 function of vaginal LCs. In previous reports, the activation of TLR3 by viral agonists and viral stomatitis virus resulted in the release of type I IFN and IFN inducible chemokines CXCL9 and CXCL11, inducing an antiviral response in LC-like DCs^47^. However, as both chemokines are potent T cell attractants, this could also lead to an influx of T cells in the tissue, indirectly increasing the risk of HIV-1 acquisition in these tissues. These findings suggest that specific TLR-induced signalling pathways and immune activation programs can render vaginal LCs susceptible to HIV-1.

We have recently identified a role for *P. timonensis*, a gram negative bacterium highly enriched in vaginal dysbiosis, in enhancing HIV-1 susceptibility. Exposure to *P. timonensis* highly increased uptake of HIV-1 by vaginal LCs and protected the virus from degradation, highlighting an important role for the vaginal microbiome in HIV-1 infection and transmission^32^. LCs are antigen presenting cells that closely interact with HIV-1 target cells (i.e. CD4 T cells) after activation and migration to a lymph node in order to direct immune responses, making them ideal first target cells for HIV-1 infection. Considering this characteristic of LCs, we have performed co-cultures of infected and extensively washed vaginal LCs with CD4 T cells to address their HIV-1 transmission capacity. Mature vaginal LCs transmitted HIV-1 efficiently to CD4 T cells, whereas immature vaginal LCs exerted their restrictive function also in these co-cultures as transmission by immature LCs to CD4 T cells was low. For freshly isolated skin-derived LCs, we have shown that langerin prevents HIV-1 transmission in immature LCs whereas mature LCs are more efficient in HIV-1 transfer due to lower langerin expression^20^. Moreover, other factors (e.g. the complement system in semen) can increase HIV-1 transmission by LCs, bypassing langerin mediated restriction^48^. Similarly, LCs found in human foreskin can become infected and transmit HIV-1 to T cells via conjugate formation^49^. However, it should be noted that the last study used virus-infected cells whereas most other studies worked with cell-free virus. Factors such as timing, viral load, cell origin and isolation methods might account for the divergence in the results. On the contrary, others suggest that langerin is involved in both uptake and transfer of HIV-1 from LCs to T cells and that langerin block prevents transmission^50^, indicating that the role of langerin is not fully elucidated yet. Moreover, immature skin LCs stimulated with Pam3CSK4 increased capture and subsequent trans-infection of HIV-1 to T cells^30^. However, the role of Pam3CSK4 in enhancing infection of target cells with RSV and HIV-1, has been suggested to be independent of TLR activation and instead a result of increased binding^51^.

While our data suggest that decreased langerin expression is involved in increased susceptibility of TLR-activated LCs, it is possible that other receptors are involved in infection and transmission. A set of receptors that might be involved in HIV-1 transmission irrespective of immune activation are Syndecans. These Heparan sulfate proteoglycans have been shown to aid HIV-1 infection of DCs^52^ as well as facilitate transmission of HCV by LCs, counteracting langerin restriction^35^. HIV-1 T/F viruses are the strains that establish infection in the genital tract during mucosal transmission^53^. Immature LCs from both skin and vaginal mucosa are significantly more susceptible to T/F viruses than chronic, laboratory adapted strains^41^. It is still not entirely clear what factors determine their superior infection^39^. T/F viruses are characterized by their higher resistance to type I IFN compared to chronic HIV-1 strains, suggesting that antiviral environments lead to selective pressure for these strains to prevail^54^.

Using primary human vaginal mucosa as well as different HIV-1 strains and isolates, we have shown that immature vaginal LCs highly expressing CD1a and langerin are poorly susceptible to HIV-1 infection and transmission in the vagina. These data indicate that immature vaginal LCs form a natural barrier against sexual HIV-1 transmission in women. Strikingly, bacterial ligands and activation of vaginal LCs increased HIV-1 infection and subsequent transmission to CD4 T cells. Thus, our study strongly suggests that bacterial pathogens can abrogate HIV-1 restriction by vaginal LCs, thereby increasing vaginal HIV-1 susceptibility. Furthermore, our data emphasize the importance for prevention of vaginal immune activation in the development of novel strategies against HIV-1/AIDS in women.

## Methods

### Tissue samples

Human vaginal tissue was collected from women undergoing vaginal surgery for pelvic organ prolapse in which excessive vaginal tissue was removed of the anterior or posterior vaginal wall. This study, including the tissue harvesting procedures, was approved by the Medical Ethics Review Committee of the Academic Medical Center (AMC) of Amsterdam. All research was performed in accordance with relevant guidelines and regulations. Vaginal tissue was freshly processed for each experiment. Surplus stroma was dissected until a thin layer of submucosa remained and tissue was cut into strips of 5–7 mm. Vaginal tissue strips were incubated overnight at 4 °C in complete medium (Iscoves Modified Dulbecco’s Medium (IMDM) of Thermo Fischer Science with L-glutamine 100 mmol/L, 10% FCS, 2500 U/mL penicillin, and 2500 mg/mL streptomycin) supplemented with Dispase II (3 U/mL, Roche Diagnostics). After incubation, the epithelial layer and lamina propria were mechanically split by the use of tweezers. Vaginal epithelial sheets were extensively washed in PBS after which was proceeded with immature LC isolation or the ex vivo culture protocol.

### Immature vaginal Langerhans cells

Epithelial sheets were cut in small pieces using surgical scissors and incubated for 10 min in PBS containing trypsin (0.05%, BD Biosciences) and DNAase I (20 U/mL, Roche Applied Science) to obtain a single cell suspension. Short incubation times (customized per donor, max. 10 min) were used to preserve cell viability and surface marker integrity. After inactivation of trypsin with FCS and thorough resuspension, cells were filtered and washed in complete medium. Further vaginal LC purification was achieved by ficoll gradient centrifugation (Axis-shield) and CD1a magnetic cell separation (MACS, Miltenyi Biotec). The resulting cell population expressed high CD1a and langerin. Cells were routinely checked for CD1a and langerin before being used for functional experiments.

### Mature vaginal Langerhans cells

Vaginal epithelial sheets were incubated in complete medium in 6-wells plates and migratory cells were collected after 3 days. Similar to immature LCs, further purification was accomplished using ficoll gradient centrifugation (Axis shield) and two rounds of CD1a magnetic cell separation (Miltenyi Biotec).

### Phenotyping LCs by flow cytometry

Immature and mature LCs were phenotyped using CD1a-APC (BD Pharmingen), langerin-PE (Novocastra), CD86-FITC (BD Pharmingen), CD80-PE (BD Pharmingen), CD83-APC (BD Pharmingen), CD4-AF488 (Biolegend), CD195 (CCR5)-PE (BD Pharmingen) and unlabeled CXCR4 (R&D systems) for which secondary detection with Goat-anti-Mouse-A488 (Invitrogen) was used. HIV-1 infection and transmission samples were stained for CD1a-APC (LC marker), CD3-PerCP (T cell marker), and p24-PE (HIV-1 envelope protein, Beckman Coulter). Immature vaginal LCs were further sorted with a FacsARIA 3 laser sorter (BD Biosciences) after staining for CD1a-APC into CD1a positive and CD1a negative fractions. Samples were analysed using FACSCanto II flow cytometers (BD Biosciences) and data analysis was carried out with FlowJo V10.

### TLR expression profile detection with RT-PCR

mRNA was isolated using mRNA Capture kit (Roche Life Sciences) and cDNA was synthesized with Reverse Transcription System (Promega). Amplification and real-time quantification was performed by PCR with SYBR Green according to manufacturer’s guidelines for the ABI 7500 Fast PCR detection system (Applied biosciences). The normalized amount of TLR mRNA (*N*_t_) was calculated from the *C*_t_ values obtained for both TLR and household (GAPDH) mRNA with the equation *N*_t_ = 2^*C*t(GAPDH)−*C*t(TLR)^.

### TLR stimulations

Immature LCs were incubated overnight in complete medium supplemented with Pam3CSK4 (5 ug/mL, Invivogen), Poly(I:C) (10 ug/mL, Invivogen), LPS (10 ng/mL, SIGMA), R848 (10 ug/mL, Invivogen), Lipid A, diphosphoryl from Escherichia coli F583 (10 ng/ml, Sigma), LPS + Polymyxin B Sulfate (PMB) (25 ug/ml, Enzolifesciences) or Lipid A + PMB (25 ug/ml) respectively. LCs were either analyzed by flow cytometry for upregulation of co-stimulatory molecule CD86, or were infected with SF162 (multiplicity of infection (MOI) 0.1).

### HIV-1 gp120 binding to langerin

TransFluorSpheres (Molecular Probes, Eugene, OR) were coated with purified HIV-1 gp120 envelope protein as described previously. HIV-1 gp120-bead-binding studies were also performed as described previously^55^. In short, vaginal LCs were pre-incubated with anti-langerin (10E2), mannan, anti-DC-SIGN (AZN-D1), anti-CD4, anti-CCR5 or left untreated, after which HIV-1 gp120-coated beads were added and binding capacity was assessed using flow cytometry.

### Cells

The langerin expressing U87 cells stably expressing CD4 and wild-type CCR5 co-receptor (obtained through the NIH AIDS Reagent Program, Division of AIDS, NIAID, NIH: U87 CD4 + CCR5 + cells) were cultured in Iscove’s Modified Dulbecco's Medium (Thermo Fischer Scientific, USA) supplemented with 10% fetal calf serum (FCS), L-glutamine and penicillin/streptomycin (10 μg/mL) as described previously^21^.

### Viruses, HIV-1 infection and transmission

R5 tropic HIV-1 strains NL4.3-Bal, JR-CSF, and SF162, were used in LC infection experiments and these viruses were generated as described previously^19^. For additional transmission experiments and infection studies using TLR ligands, SF162 and NL4.3-Bal virus was used. Moreover, the HIV-1 Transmitted/Founder (T/F) virus CH058 was used for transmission from immature vaginal LCs to U87. CH058 was generated as previously described^41^. Immature and mature LCs were infected with a MOI of 0.1 and HIV-1 infection was assessed by flow cytometry at day 5 after infection by intracellular p24 staining. To determine transmission of HIV-1 from LCs to T cells, infected LCs were washed extensively at day 2 of infection and were subsequently co-cultured with allogeneic PHA/IL-2 activated CD4 T cells for 3 days. Infection levels in CD4 T cells were measured to assess HIV-1 transmission by vaginal LCs. Additionally, vaginal immature LCs were stimulated with LPS for 24 h (NL43Bal) or 30 min (CH058) before infection. After 2 days, cells were washed extensively and co-cultured with U87 to determine transmission to these target cells. After 3 days of co-culture, vaginal LCs were washed away and infection in U87 cells was measured by flow cytometry.

## Supplementary Information

Supplementary Figure 1.

## Data Availability

The datasets generated during and/or analysed during the current study are available from the corresponding author on reasonable request.
