# Supplementary material for: Immune activation of vaginal human Langerhans cells increases susceptibility to HIV-1 infection
Source: Sci Rep. 2023 Feb 25;13:3283. doi: 10.1038/s41598-023-30097-x (PMC9968315; doi:10.1038/s41598-023-30097-x)
Supplement: Supplementary file 1 — Supplementary Figure 1. [file 41598_2023_30097_MOESM1_ESM.pdf]

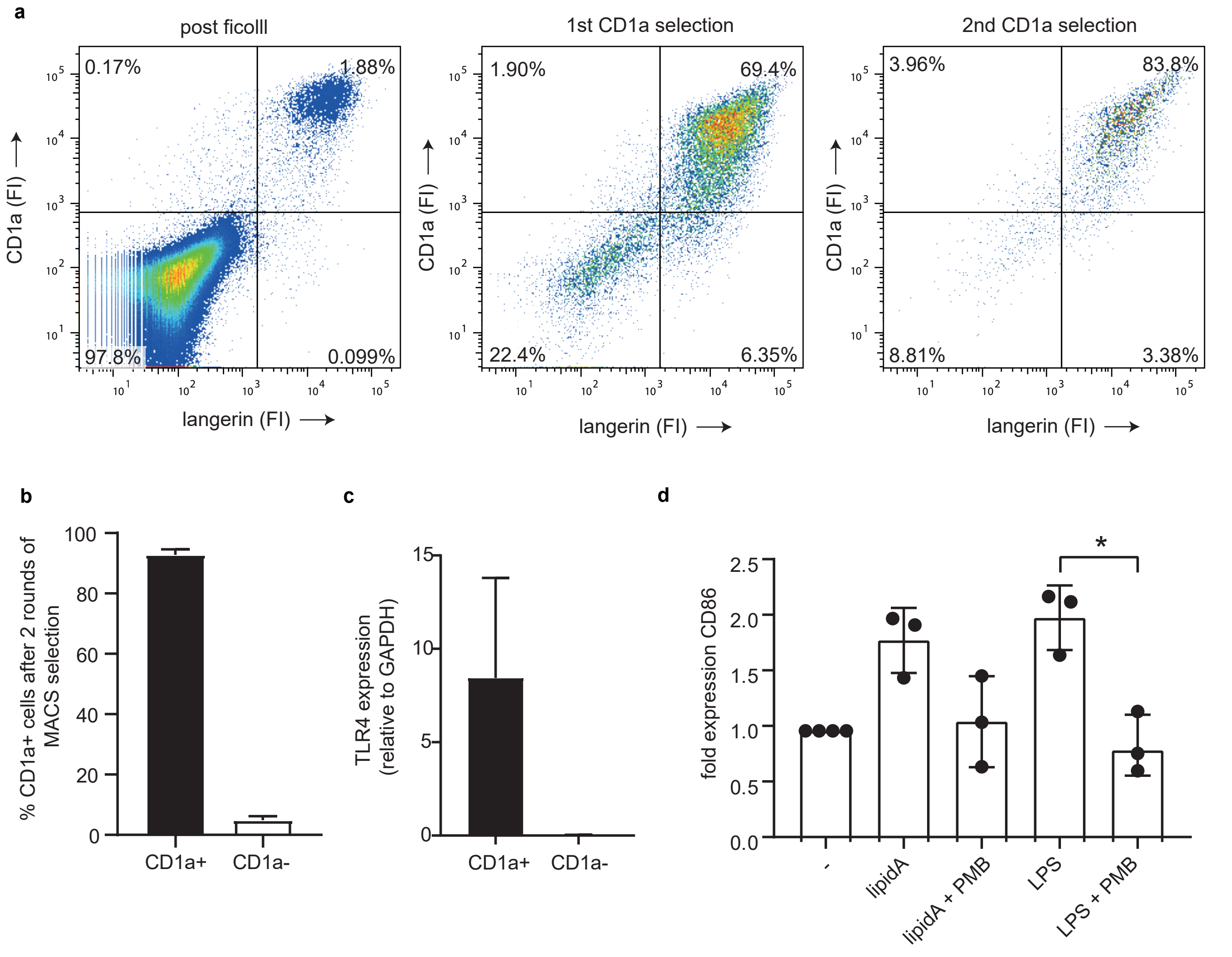


**Supplemental figure 1.:**
(**a**, one representative donor) CD1a and langerin expression of cells isolated from vaginal mucusa after ficoll, 1st CD1a MACS selection, 2nd CD1a MACS selection. (**b**, N=8) improved isolation of CD1a positive immature vaginal LCs after two MACS selection steps. (**c**, N=4) TLR4 expression on immature vaginal LCs after sorting into CD1a positive and CD1a negative fractions. Expression was determined by real time quantitative PCR and values are depicted normalized to GAPDH. (**d**, N=3) expression of CD86 on immature vaginal LCs after stimulation with Lipid A, LPS, PMB or a combination thereof. * p <0,05, two-tailed t-test; data are mean ± SD.
